# Fermentative Production and Thermostability Characterization of **α** Amylase from *Aspergillus* Species and Its Application Potential Evaluation in Desizing of Cotton Cloth

**DOI:** 10.4061/2011/323891

**Published:** 2011-10-03

**Authors:** Murali Krishna Chimata, Chellu S. Chetty, Challa Suresh

**Affiliations:** ^1^Department of Biotechnology, Acharya Nagarjuna University, Guntur 522 510, India; ^2^Department of Natural Sciences, Savannah State University, Savannah, GA 31404, USA; ^3^Department of Biochemistry, National Institute of Nutrition, Tarnaka, Hyderabad 500 007, India

## Abstract

The production of extracellular amylase was investigated employing our laboratory isolate, *Aspergillus niger* sp. MK 07 and effect of process variables on enzyme production, was studied in a fermentor. It was found that amylase production was maximum when the fermentor volume was maintained at 70%, rate of agitation at 250 rpm, air supply at 2.5 vvm, inoculum concentration of 10%, and a pH of 5.0. Highest enzyme production obtained under all optimized conditions was 1734 U/mL with sucrose as carbon substrate and corn steep liquor as nitrogen source. Enzyme purification studies by ammonium sulphate precipitation and Sephadex G-100 chromatography was evaluated for obtaining purified enzyme. Thermostability of amylase were evaluated with varying concentrations from 0.2 to 0.5 M concentrations of calcium chloride and the highest activity obtained was 3115 U/mL with 0.3 M calcium chloride at 55°C. Effect of temperature and pH on the activity of purified enzyme was evaluated and the purified enzyme showed an activity till 75°C and a pH of 6.5. Application potential of partially purified alpha amylase on desizing of cotton cloth was evaluated with varying enzyme concentrations from 50 to 500 U/mL and the highest desizing activity was found to be at 300 U/mL.

## 1. Introduction

Microbial enzymes are widely used in several industries, notably in detergent, food processing, brewing, and pharmaceuticals [1]. In fact, their use has been recorded since ancient times without knowing the functional utility in oriental countries [2]. They are also used for diagnostic, scientific, and analytical purposes [3]. In the present era, enzymes such as proteases, glucoamylases, glucose isomerase, and pectinases became part of our daily life and are extensively used as commodities [4]. In the present biotechnological era, microbial amylases have been investigated for their role in the manufacturing of maltose and also in the manufacturing of high-fructose corn syrup [5]. Amylases are also used in manufacture of oligosaccharide mixture, manufacture of maltotetrose, manufacture of high-molecular-weight branched dextrins, removal of starch sizer from textiles, and so forth [1, 6]. Amylases are amongst the most studied enzymes [7]. Their enormous diversity of function makes them one of the most fascinating groups of enzymes for application at different sectors of life in both physiological and commercial fields [8]. This vast diversity of amylases, in contrast to the specificity of their action, attracted worldwide attention in attempts to exploit their physiological and biotechnological applications [9]. 

Amylases are starch-degrading enzymes that catalyze the hydrolysis of internal alpha 1-4 glycosidic bonds in polysaccharides with the retention of alpha anomeric configuration in the products [10]. Amylases from plants and microbial sources have been employed for centuries in brewing industry [11]. Fungal amylases are widely used for the preparation of oriental foods [12]. Amylases of bacteria, fungi, and viruses are increasingly studied due to the relative ease of large-scale production (low downstream cost as they are extracellular in nature) as compared to amylases from plants and animals and their importance in subsequent application at industry [13]. In addition, bacterial amylases have longer shelf life and can be stored for weeks without significant loss of activity [14]. Extracellular amylases are important for the hydrolysis of starch and cellulose in cell-free environments and enable the cell to absorb and utilize hydrolytic products [15]. Amylases are one of the most important industrial enzymes that have a wide variety of applications ranging from conversion of starch into sugar syrups, to the production of cyclodextrins for the pharmaceutical industry [8]. These enzymes account for about 30% of the world's enzyme production. The overall potential of amylases in industrial process is yet to be exploited fully. The inherent disadvantages in the use of amylase, in particular, are related to the complete cost of enzyme production, and downstream processing is one of the major obstacles against the successful application of any technology in the enzyme industry [16]. Thermal, operational, and storage problems occur as they are easily prone to inactivation by self-degradation (autolysis), whereas good industrial catalyst should be stable under the toughest operating conditions and for long durations [11]. 

To overcome such limitations, great attention has been devoted for studies on amylases to tackle the problem [3]. Recent approaches for increasing amylase yield including screening for naturally occurring enzymes with intrinsic stability or to produce stable enzymes by means of protein engineering and optimization of fermentation media through a statistical approach are some of them [17]. The simplest approach to obtain a stable enzyme is to look for the desired enzyme in a readily available organism [18]. Hence great interest has been generated in the search for new thermophilic, alkaliphilic strains and their fermentation conditions optimization by using statistical methods to get more economical yields for industrial applications [19]. The success of fermentation greatly depends on the use of right type of organism that can produce the desired product at minimum cost and in large quantities [4]. 

The overall objective of the present study was to isolate a novel *Aspergillus *species which has got the inherent capacity to produce alpha amylase and to develop a strategy for the fermentative production at higher scale by optimizing all the essential fermentative kinetics and other aspects which can result in increase in yield or productivity of the enzyme production and to study the effect of calcium chloride on the thermostability of alpha amylase and also to investigate the optimum amylase enzyme concentration on desizing of cotton cloth by partially purified enzyme.

## 2. Materials and Methods

### 2.1. *Aspergillus* Strain

Isolation of the *Aspergillus* strains was carried out with various soil samples collected from dump yards of a local starch plant near Hyderabad. *Aspergillus* species were identified by standard blotter method [20]. They were identified on the basis of morphological characteristics and certain standard confirmatory tests according to Bergey's manual of Bacteriology.

### 2.2. Isolation of the Strain

The soil suspension was diluted up to 10^−3^–10^−6^ times, and 0.5 mL of each diluted suspension was then transferred by spread plate method with a sterile glass spreader on petri plates containing potato dextrose starch agar medium (PDSA). These petri plates were incubated at 30°C for 4-5 days. Based on the zone of clearance on the starch agar plates, 13 colonies were picked up, and individual amylase activity of selected colonies was, investigated carried out and strain MK 07 showing good activity was used for further studies. The young colonies of fungal cultures were aseptically picked up and transferred to potato dextrose starch agar slants with 1% starch. These slants were then incubated at 30°C for 4 days, and after sufficient growth, they were stored at 4°C in the refrigerator till further use.

### 2.3. Inoculum Preparation

Actively growing and heavily speculating ten-day-old potato dextrose starch agar slant culture was added to 10 mL sterile 0.85% sodium chloride salt solution. The spores were gently scraped off with the help of a sterile needle, and contents were passed through glass wool so as to obtain spore inoculums free from mycelial bits. A volume of one mL of spore suspension contained more than 2.6 × 10^6^ spores.

### 2.4. Measurement of Amylase Activity

The activity of the alpha amylase was determined by the Bernfeld [21] procedure using soluble starch (Sigma chemical, USA) as a substrate. The reaction mixture containing 1 mL of 1% substrate (w/v) in 0.02 M phosphate buffer (pH 6.8), 0.5 mL of crude enzyme, and 0.5 mL of the buffer was incubated for 5 mins at 55°C. The reaction was stopped by adding 2 mL of a solution of 3, 5-dinitro salicylic acid (DNS), followed by cooling to room temperature. The concentration of the reducing sugar was measured at 540 nm in an UV-Vis spectrophotometer using maltose as standard. The blank contained 0.5 mL, 0.02 M phosphate buffer (pH 6.8), 0.5 mL crude enzyme, and 1 mL of 1% starch solution. One unit (U) of alpha amylase is defined as the amount of enzyme that releases 1 micromole of reducing sugar as maltose per minute under the assay condition and is expressed as U/mL of substrate in submerged fermentation and other studies [22].

### 2.5. Stock Solution

Maltose was used as standard reference for amylase activity. One mg/mL maltose solution was prepared and used as stock solution. Ten appropriate dilutions from 0.1 to 1.0 were made from standard stock solution. One mL of each dilution and 1 mL of DNS solution was added in each test tube, and blank was made with 1 mL of distilled water and 1 mL of DNS solution. These tubes were placed in boiling water bath for 5 mins and cooled to room temperature. The contents of the test tubes were diluted up to 10 mL with distilled water. All the tubes were mixed well, and optical density of the solution was measured at 540 nm. A standard curve was constructed taking concentration of maltose (mg/mL) on *x*-axis and corresponding optical density on *y*-axis [23].

### 2.6. Protein Estimation

The protein content of the enzyme preparations was estimated by the Lowry method using Bovine serum albumin as standard. One mg/mL stock solution is prepared, and from that stock solution various dilutions ranging from 0.1 to 1.0 mg/mL were prepared, and standard plot was performed. From each dilution, 0.2 mL of solution was taken in different test tubes, and to each tube 2 mL was added of alkaline copper sulphate reagent, and this mixture was mixed well and incubated at room temperature for 10 mins. Then 0.2 mL of the Folin-Ciocalteu reagent was added to each tube and incubated for 30 mins. After incubation, absorbance was read at 595 nm in an UV-Vis spectrophotometer. Then a standard graph was plotted with concentration on *x*-axis and optical density on *Y*-axis [21, 22].

### 2.7. Extraction of the Enzyme

After 48 hrs of incubation period, the culture broth from fermentor was collected in a sterile conical flask. To 250 mL of culture broth, 100 mL of phosphate buffer (0.02 M) and 0.02% tween 80 was added. The contents in the flasks were equally distributed into 250 mL centrifuge tubes, and these tubes were centrifuged in the centrifuge machine at 5000 ×g for 30 minutes. The substrate-free suspension was used for the estimation of alpha amylase [23].

### 2.8. Reactor Studies

The isolated *Aspergillus niger *strain MK 07 was grown in 2 L B-Braun fermentor (Germany) containing one litre of medium consisting of synthetic media and glucose as a carbon source in varying ranges from 10 to 40 g L^−1^ at pH 5.0 by incubating at 35.0°C and at 150 rpm for 12 h. Growth was measured by estimating the absorbance at 600 nm. The dry weight of cell mass was estimated from a previous calibrated curve of absorbance versus cell mass according to [24]. The cell-free supernatant was used to measure glucose concentration and amylase activity. All assays were carried out in triplicate.

### 2.9. Enzyme Purification

All enzyme purification steps were carried out at 0 to 4°C.

#### 2.9.1. Ammonium Sulphate Precipitation

The crude broth obtained after fermentation was centrifuged at 5000 ×g for 30 mins to remove the cell biomass. Solid ammonium sulphate was added slowly to the culture supernatant to get 60% saturation, stirred for 60 min, and left for overnight at 4°C. The precipitate was harvested by centrifugation at 10,000 ×g for 10 min, dissolved in 50 mM glycine-sodium hydroxide buffer and dialyzed against same buffer overnight (4°C). The dialyzed sample was then assayed for amylase activity and glucose content [23].

#### 2.9.2. Sephadex G-100 Chromatography

Dialyzed enzyme was loaded on to a column of sephadex G-100 (1.5 × 90 cm) previously equilibrated with 50 mM glycine-sodium hydroxide buffer (pH 11) and then eluted at a flow rate of 10 mL/h with the same buffer containing sodium chloride gradient from 0.1 to 1 M, and 1 mL volume fractions were collected. The absorbance of fractions was checked at 600 nm. Those fractions that showed absorbance were assayed for amylase activity with starch as substrate. Amylase-active fractions were pooled and concentrated for further characterization [25].

### 2.10. Enzyme Characterization

#### 2.10.1. Determination of the pH Optimum and pH Stability

The pH optimum for purified amylase was assayed by analyzing its activity in the pH range of 5 to 12 using starch as a substrate and buffer systems of 0.05 moL L^−1^ phosphate buffer for pH 5.0 to 7.5, tris-HCl for pH 8.0 to 9.0, glycine-sodium hydroxide for pH 9.5 to 11.0, sodium phosphate for 11.5 to 12.0, and sodium carbonate for 12.5 to 13.0. pH stability study of the protein was analyzed by preincubating 5 mL of purified enzyme in 3.5 mL of selected pH buffer at 37°C for 1 to 48 h and subsequent analysis of residual activities under standard assay conditions.

#### 2.10.2. Determination of Optimum Temperature and Thermal Stability

To study the temperature optima of enzyme, the enzyme reaction mixture was incubated at different temperatures ranging from 35°C to 75°C in glycine-sodium hydroxide buffer (pH 11.0), and amylase activity was measured using starch (1%) solution as substrate. For determining thermal stability, the enzyme was preincubated for 1.0 h at different temperatures ranging from 35 to 90°C, and the relative activity was measured under standard assay conditions after incubating with starch as substrate [26].

#### 2.10.3. Thermal Inactivation Studies

Thermal inactivation assays were carried out by preheating 950 *μ*L of standard buffer at the corresponding temperature, then adding 1 *μ*g protein in 50 *μ*L of the same buffer and preincubating the mixture at the same temperature. Samples were collected every 1.0 h at 75, 80, 85, and 90°C and cooled to 70°C before analyzing the amylase activity.

### 2.11. Amylase Application Potential Evaluation

#### 2.11.1. Alpha Amylase as Desizer

 A clean and stiffed cloth with starch over that was used for the study. Equal sizes of pieces were used for the study, and they were weighed on an electric balance (5 × 5 inch). The cloth strip was then dipped in 100 mL of enzyme solution (pH 6.5) and then placed in the incubator at 70–80°C for 1.0 hr. After the time interval, the cloth strip was washed with tap water and then oven-dried. After drying, the cloth strip was again weighed.

(1) Weight  of  starch  removed=initial  weight−final  weight.

The % removal of starch was calculated by applying the following formula:

(2) $$\begin{array}{c} \text{\%}\;\text{of}\;\text{desizing}=\frac{\text{Wt}\;\text{of}\;\text{starch}\;\text{removed}\;\text{by}\;\text{enzyme}}{\text{Total}\;\text{starch}\;\text{present}\;\text{on}\;\text{the}\;\text{cotton}\;\text{strip}}.\; \end{array}$$

Total starch was calculated by hydrolysing the starch with 0.1 N sulphuric acid.

The following parameters were studied for desizing of the cotton cloth.

#### 2.11.2. Effect of Enzyme Concentration

The effect of enzyme concentration on the desizing of the cotton cloth by crude and partially purified enzyme was investigated. The concentration of the enzyme was varied from 100 to 500 U/mL/min.

#### 2.11.3. Effect of Temperature

 The effect of temperature (25–50°C) on the desizing of cotton cloth by crude and partially purified enzyme was studied.

#### 2.11.4. Effect of pH

The effect of different pH (4.0–9.0) on the desizing of cotton cloth by crude and partially purified enzyme was studied.

#### 2.11.5. Time Profile for the Desizing of the Cotton Cloth

The rate of desizing of cotton cloth at various time intervals by crude and partially purified alpha amylase was estimated. The cloth was treated with crude and partially purified enzymes at 80°C for 75 mins.

## 3. Results and Discussion

### 3.1. Fermentor Study

Kinetic study and volumetric rates of enzyme formation and biomass revealed that the mediums containing sucrose as carbon source gave better results compared to other medium tested. The medium used for amylase production consists of (g/L) sucrose 30; corn steep liquor 20; magnesium sulphate 0.5; potassium chloride 0.5; potassium phosphate 1; ferrous sulphate 0.01; peptone 5. Hence further fermentor studies were carried out with the above-mentioned medium for amylase production by the isolated *Aspergillus niger* MK 07. 

The following parameters were evaluated and optimized for amylase production by the isolated *Aspergillus niger* MK 07 strain.

#### 3.1.1. Effect of Fermentor Media Volume

**Figure 1:**
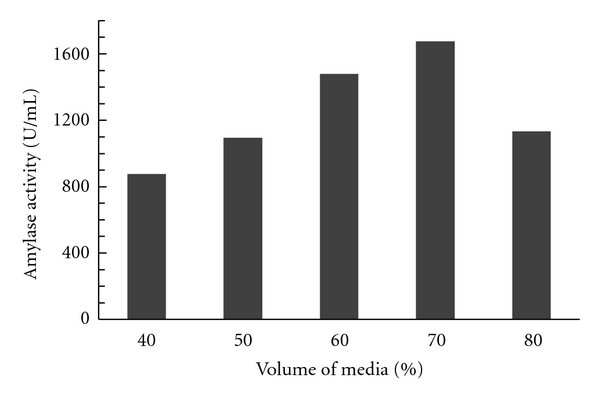
Effect of volume of media on amylase activity.

The effect of fermentor volume on the production of alpha amylase by isolated MK 07 strain was evaluated. Maximum production of 1675 U/mL was achieved when the reactor volume was kept at 70%. As the volume of the fermentor media was increased, the enzyme production was decreased. The kinetic values of Y p/x, and Q_p_ were also found to be significant when the volume of the fermentor was maintained at 70%. When the medium volume percentage in the fermentor was increased to 80%, a decrease in amylase production was obtained. Most of the fermentation studies for enhanced amylase production were carried out by synthetic media [23]. Hence, for further studies, the volume of the media was maintained at 70% as shown in Figure 1.

#### 3.1.2. Effect of Varying Rate of Agitation

**Figure 2:**
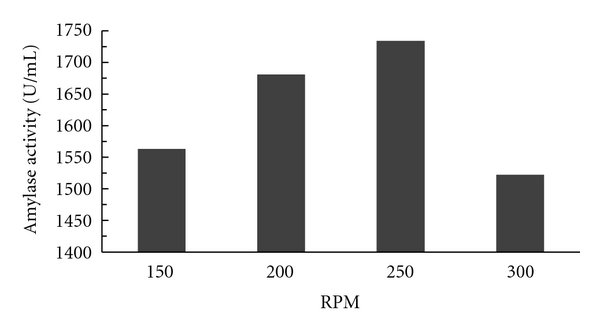
Effect of different RPM on amylase activity.

Effect of varying rate of agitation was investigated for alpha amylase production in fermentor by the isolated strain. The fermentation was carried out at 150, 200, 250, and 300 rpm. The production of enzyme following growth of the organism was found to be maximum of 1734 U/mL at an rpm of 250. When the agitation rate of the fermentation broth was increased above 250 rpm, a decrease in enzyme production was observed. Hence, for further studies, the rpm was maintained at 250 as shown in Figure 2.

#### 3.1.3. Effect of Different Volume of Air Supply

**Figure 3:**
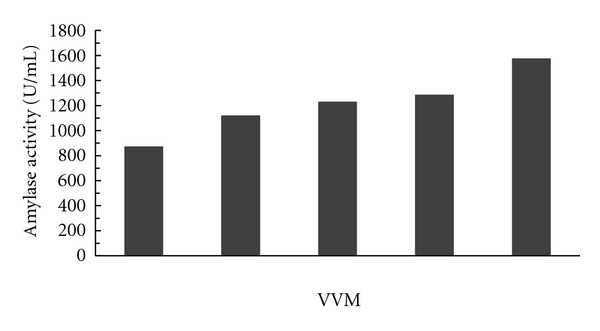
Effect of air supply on enzyme activity.

Effect of different range of air supply (0.5–2.5 vvm) to the fermentation medium for the production of alpha amylase by the isolated strain MK 07 was studied. The production of alpha amylase enzyme following growth of the organism was optimum and maximum at 2.5 vvm (1576 U/mL), and further increase or decrease in air supply decreased the enzyme production. The production of amylase enzyme steadily increased with increase in air supply, indicating the isolated strain is an aerobic organism as shown in Figure 3.

#### 3.1.4. Effect of Different Inoculum Sizes

**Figure 4:**
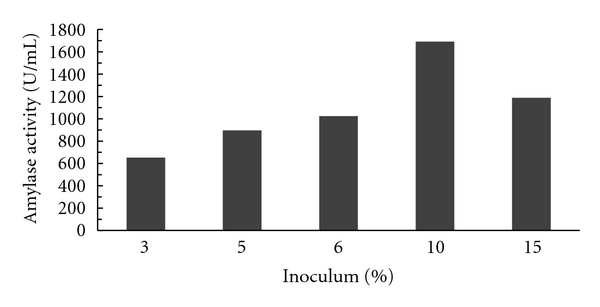
Effect of different inoculum percentages on enzyme activity.

Effect of different sizes of inoculum on the production of alpha amylase by the isolated strain MK 07 was investigated. The production of enzyme following growth of the organism was increased with increase in inoculum volume up to 10%, and upon further increase in inoculum volume, enzyme production decreased, and the maximum amylase production obtained was 1691 U/mL as shown in Figure 4.

#### 3.1.5. Effect of pH

**Figure 5:**
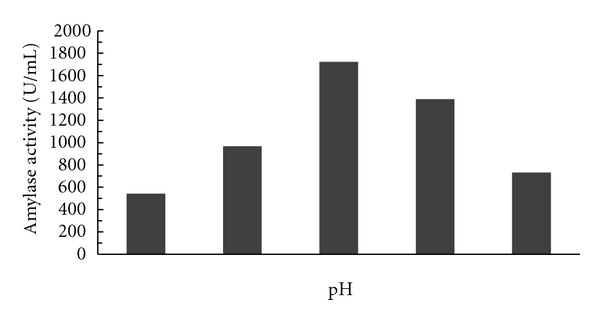
Effect of different pH on the activity of amylase.

Effect of different pH on the production of alpha amylase by the isolated strain Mk 07 was investigated. The production of enzyme increased with increase in pH up to 5.0, and upon further increase in pH, the enzyme production decreased drastically, and the maximum amylase production obtained at a pH of 5.0 was 1723 U/mL, and the results are shown in Figure 5. 

Under all optimized conditions a high enzyme production of 1723 U/mL was obtained in 48 hrs.

### 3.2. Effect of Calcium Chloride on the Thermostability of Alpha Amylase

**Figure 6:**
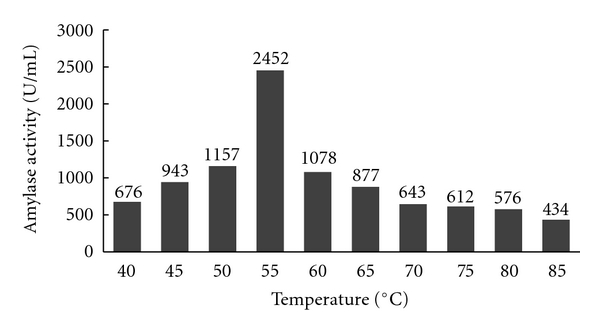
Effect of 0.2 M calcium chloride concentration on amylase activity.

**Figure 7:**
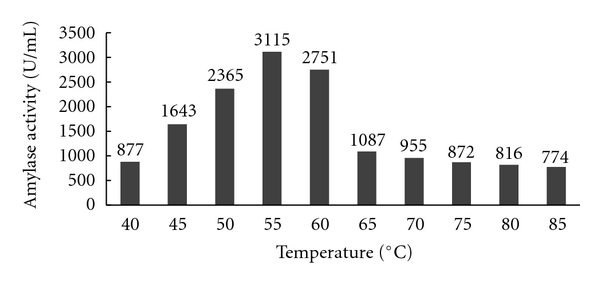
Effect of 0.3 M Calcium chloride on amylase activity.

**Figure 8:**
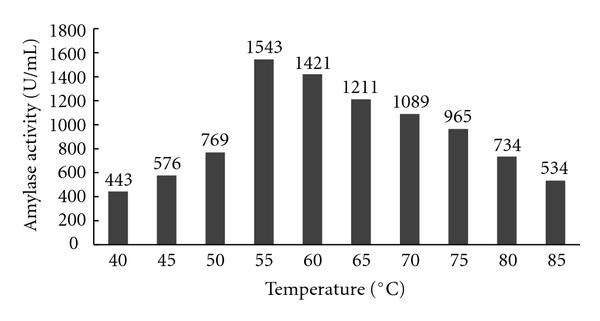
Effect of 0.4 M Calcium chloride on amylase activity.

**Figure 9:**
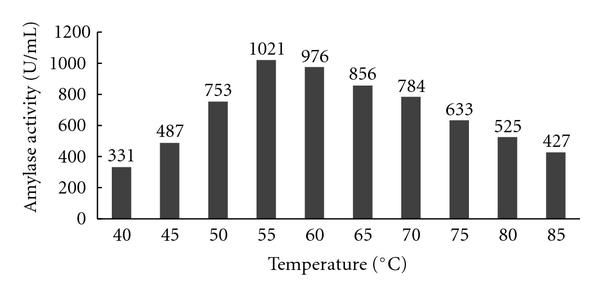
Effect of 0.5 M Calcium chloride on amylase activity.

Different concentrations of calcium chloride like 0.2, 0.3, 0.4, and, 0.5 M were added to the fermentation medium and the enzyme produced by the *Aspergillus* species was tested for the thermostability of alpha amylase. The thermostability of the crude enzyme was increased with the increase in the concentration of calcium ion. The enzyme produced by *Aspergillus* species was found to be most thermostable at 50–60°C at 0.3 M concentration of calcium chloride. Further increase in the concentration levels decreased the thermostability of the enzyme. The results of the above experiments are shown in Figures 6, 7, 8, and 9.

### 3.3. Partial Purification of Alpha Amylase

The alpha amylase was partially purified by ammonium sulphate precipitation and Sepahdex G 100 × chromatography. The specific activity of the enzyme was gradually increased after purifying the alpha amylase and found optimum after ammonium sulphate precipitation. The specific activity of the enzyme was found to increase after ammonium sulphate precipitation (see Table 1).

#### 3.3.1. Effect of Temperature on the Activity of the Enzyme

**Figure 10:**
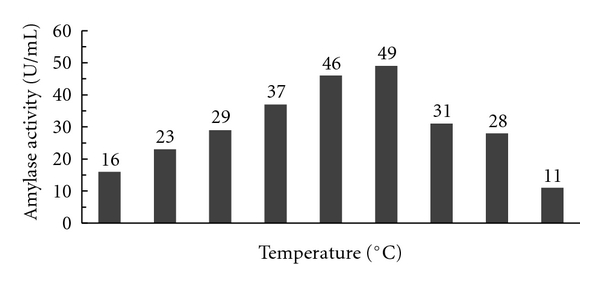
Effect of temperature on enzyme activity.

The residual activity of the partially purified alpha amylase enzyme was measured by incubating the enzyme at different temperatures. The results showed that the activity of the enzyme increased with increase in temperature. The partially purified enzyme activity was found to be highly active between 70–75°C. As the optimum activity was obtained at 75°C, thus this temperature was optimized for the conversion of starch to oligosaccharides, and the results of the following experiment are shown in Figure 10.

#### 3.3.2. Effect of pH on the Activity of the Enzyme

**Figure 11:**
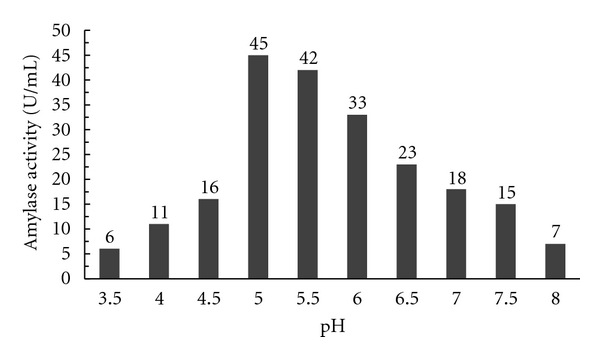
Effect of pH on enzyme activity interns of starch hydrolysis.

The residual activity of the partially purified alpha amylase enzyme was measured by incubating the enzyme at different pH. The results showed that the activity of the enzyme increased with increase in pH. The partially purified enzyme activity was found to be highly active between pH 5.0 and 5.5. As the optimum activity was obtained at pH 5, thus this pH was optimized for the conversion of starch to oligosaccharides, and the results of the above study are shown in Figure 11.

### 3.4. Alpha Amylase as Desizer

#### 3.4.1. Effect of Enzyme Concentration

**Figure 12:**
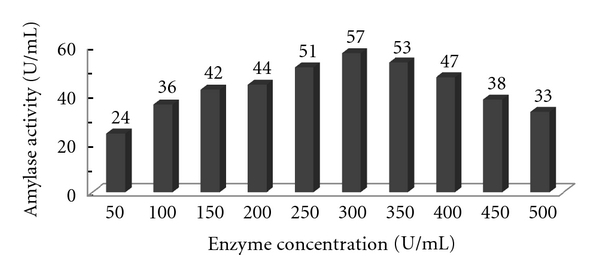
Effect of enzyme concentrations on the desizing of cotton cloth by partially purified alpha amylase.

The effect of enzyme concentration on the desizing of the cotton cloth was studied by partially purified enzyme. The concentration of enzyme was varied from 50 to 500 U/mL/min. The desizing (separation of starch from the cloth) of the cloth was increased with increase in enzyme concentration and was found to be optimum at 300 U/mL/min. Further increase in the concentration had no significant effect on the desizing of the cloth. The results of the above experiment are shown in Figure 12.

## 4. Discussion

The present investigation was aimed at achieving maximum enzyme production systematically by optimizing various process variables which have a significant role in the determination of growth of the strain as well as production of enzyme [27]. Overall evaluation of criterion-based optimization of enzyme production was performed assigning the relative weightage to reusability and industrial applicability. The data suggested that carbon substrate plays very significant role apart from pH, agitation, and air supply [28]. Most of the fermentation studies for enhanced amylase production were carried out by synthetic media [26]. Agitation intensity influences the mixing and oxygen-transfer rate in many fungal fermentations thereby influencing mycelial morphology and product formation [14]. Agitation intensities of up to 300 rpm have normally been reported in the literature for the production of amylase from various microorganisms [27]. Kinetic study revealed the values of product yield coefficient (Y p/x), volumetric rate of enzyme (Q_p_), biomass formation (Q_x_), and specific rate of product formation (qp). Enzyme production was found maximum when the agitation intensity was maintained at 250 rpm. At low level of inoculum percentage, the production of enzyme was insignificant and at higher levels of inoculum percentage did not have any considerable increment in enzyme production. Under optimized inoculum sizes, the amylase production increased, and an increase in biomass was also observed. Reference [22] reported highest enzyme production with an inoculum level of 10% (v/v), and the maximum amylase production obtained was 281 U/ml. Reference [23] has reported that no morphological changes occur in *Aspergillus* strain in air-life bioreactors and that pellet size decreases considerably when the air velocity is increased. Among the physical parameters, the pH of the growth medium plays an important role by inducing morphological change in the organism and in enzyme secretion. The pH change observed during the growth of the organism also affects product stability in the media [2, 7, 27]. Various other researchers have reported that a pH of 6 was optimal for amylase production by *Bacillus strains* which are used commercially. A lot of work on the morphology and physiology of alpha amylase production by *Aspergillus strain* during batch cultivation has been done [22]. Evaluation of varying concentrations of calcium chloride on the thermostability of alpha amylase revealed that the thermostability of crude enzyme increased with increase in molar concentration of calcium chloride up to a certain extent, and further increase in concentrations had no effect on the thermostability, and upon further increase of concentrations, thermostability decreased. The results of the present study showed increased enzyme production on optimization of various process parameters as well as effective desizing of cloth removal property of the enzyme alpha amylase for which *Aspergillus* strain is one of the principal sources.

## Figures and Tables

**Table 1:** Partial purification of alpha amylase from fermented broth.

Step	Amylase activity (U/mL)	Protein (mg/mL)
Fermentation broth	327	3.75
Ammonium sulphate precipitation	1427	1.63
Sephadex G 100 Chromatography	3286	0.73
